# Transitioning enzyme catalysis towards photocatalysis

**DOI:** 10.1098/rsta.2023.0380

**Published:** 2025-05-08

**Authors:** Nigel Scrutton, Sam Hay, Derren Heyes

**Affiliations:** ^1^ Department of Chemistry, The University of Manchester, Manchester, UK

**Keywords:** photocatalysis, enzyme catalysis, photobiocatalysis, photoenzyme

## Abstract

Enzyme biocatalysis is being industrialized at a phenomenal rate. Biocatalysis offers routes to chemical transformations that avoid the use of expensive metal catalysts, high temperatures and pressures, while providing impressive enantio-, regio- and chemo-selectivities. Working individually or as cascades, in live cells or cell-free preparations, to manufacture everyday chemicals, materials, healthcare products, fuels and pharmaceuticals and in diagnostic and industrial sensing applications, enzymes are key enablers in a circular bioeconomy. An ability to exploit and tailor biocatalysts rapidly and predictably requires knowledge of structure-mechanism relationships and the physical chemistry of enzyme action. This knowledge has advanced since our millennium article on this topic (Sutcliffe and Scrutton *Phil Trans R. Soc. Lond. A*. 2000. 358, 367–386). Here, we discuss an emerging frontier—enzyme photobiocatalysis. Photoenzymes are rarely found in nature. This limits 'difficult-to-achieve' reactions in biology that are generally accessible to chemical photocatalysts. We discuss here the emergence of photobiocatalysis as a new frontier. We review knowledge of natural photoenzymes and identify challenges and limitations in their use as photobiocatalysts. We consider emerging reports on repurposing natural enzymes as photobiocatalysts. We also discuss prospects for de novo design of photobiocatalysts which as a general concept would transform catalysis science.

This article is part of the theme issue ‘Science into the next millennium: 25 years on’.

## 1. Introduction

Enzymes are a cornerstone of the bioeconomy. They are central components of technologies underpinning the circular economy and offer engineering biology routes to realizing global challenges, including net zero, clean growth and the establishment of the bioeconomy. An ability to exploit and tailor enzyme activities both rapidly and predictably is essential to realizing these contemporary global challenges. The vast majority of natural and engineered enzymes are thermally activated. This often limits their use to those reaction types found naturally in biology, although there are notable exceptions. It also places a high dependence on expensive and unstable cofactors and coenzymes and a sizeable demand on the provision of energy sources (e.g. biochemical or use of artificial reductants).

The full potential of biocatalysis has yet to be unlocked. Accessing new chemistries and expanding the scope of existing reactions is essential to make biocatalysis a pivotal technology across the whole industrial manufacturing spectrum. There have been many innovations to overcome limitations in the application of enzymes, including but not limited to (i) the construction of artificial metalloenzymes to combine biological and chemo-catalytic components [1], (ii) the incorporation of non-canonical amino acids to escape inherent chemical limitations of the proteinogenic amino acids [2], (iii) triggering new reactivities through the transfer of organocatalytic modes to enzymes [3], and (iv) the de novo design of enzymes [4]. With notable exceptions, missing from this list are photobiocatalysts, which are needed more generally to access ‘difficult-to-achieve’ reactions. To date, generalized platforms for photobiocatalyst discovery have not been established. This is urgently needed to enable the rapid engineering of photobiocatalysts to access new and difficult-to-achieve reaction types with broad reaction scopes.

The use of light to drive enzyme catalysis bypasses many hurdles associated with the use of conventional, thermally activated enzymes. Nature generally does not make use of enzymatic photocatalysis as there are only three known, natural photoenzymes. Therefore, biology cannot access a broad range of difficult-to-achieve reactions that would be transformational in catalysis science and the application of these reactions in the modern world. Light is freely available and non-invasive, yet the photochemical versatility of natural cofactors (e.g. flavin [5]) is seldom used by enzymes. Therefore, securing generalized routes to the predictive design of photobiocatalysts is a fundamental biological challenge. Identifying rules for and setting out generalized routes to the design of photobiocatalysts would be transformative both in catalysis science and for the emerging bioeconomy.

Predictive photobiocatalysis would ‘open up’ new high-energy reaction pathways, while maintaining the beneficial, highly selective, specificities of thermally activated enzymes. It would also generalize photobiocatalysis, giving broad access to difficult-to-achieve reactions, through top-down protein engineering and bottom-up de novo design, informed by measurement and predictive modelling. This would bring photobiocatalysis into the biological mainstream—releasing it from its current Cinderella position—by expanding reaction space and substrate scope well beyond that available with natural or engineered thermally activated enzymes.

In this review, we provide a perspective on, and an emerging prospective view of, biological photocatalysis. Our perspective is informed from structural-mechanistic and computational studies of natural photoenzymes. Our prospective view then builds on this framework to include early insight into photobiocatalyst design and engineering. This is achieved through the top-down repurposing of thermally activated enzymes and natural photoenzymes, or from the bottom-up de novo design of new photoenzymes.

## 2. Experimental probes of enzyme catalysis

The framework typically used to describe enzyme kinetics, and discussed extensively in our previous Millennium article [6], is transition state theory. The activation free energy (barrier) is obtained from the temperature dependence of the measured rate constant(s) via the Eyring equation:


(2.1) $$k=\kappa \frac{\;k_{B}T}{h}exp\left(-\Delta G^{‡}/RT\right).$$

This allows the activation enthalpy ($\Delta H^{‡}$) and entropy ($\Delta S^{‡}$) to be determined in the usual way ($\Delta G^{‡}=\Delta H^{‡}-T\Delta S^{‡}$). Alternatively, the Arrhenius equation is often used in place of equation (2.1) to determine the apparent activation energy (*E*_a_) and prefactor (*A*) terms:


(2.2) $$k=A\;exp\left(-E_{a}/RT\right).$$

More recently, the temperature dependence of $\Delta H^{‡}$ and $\Delta S^{‡}$ have been investigated by including a (temperature independent) heat capacity term, $\Delta C_{p}^{‡}$, which can be used to expand equation (2.1):


(2.3) $$\begin{array}{rl} \Delta S^{‡} & =\Delta S_{T_{0}}^{‡}+\Delta C_{p}^{‡}\;ln\left(T/T_{0}\right),\;\\ \Delta H^{‡} & =\Delta H_{T_{0}}^{‡}+\Delta C_{p}^{‡}\;\left(T-T_{0}\right).\; \end{array}$$

**Figure 1.**
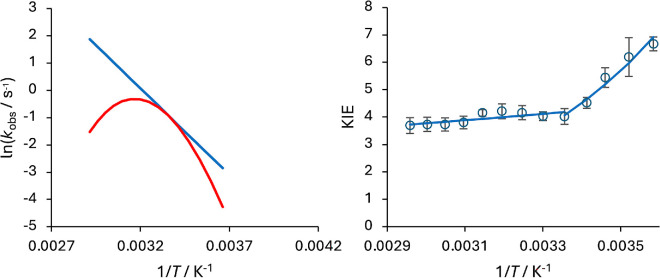
*Left*: The temperature dependence of typical enzyme-catalysed reactions demonstrating the effect of negative activation heat capacity. The reactions are modelled using the Eyring equation (2.1) with $\Delta H^{‡}$ = 50 kJ mol^−1^ and $\Delta S^{‡}$ = −100 J mol^−1^ K^−1^. The red data also include an activation heat capacity contribution, $\Delta C_{p}^{‡}$ = −3 kJ mol^−1^ K^−1^ (equation 2.3). *Right*: Example of an unusual temperature dependence of a primary kinetic isotope effect (KIE), *k*^H^/*k*^D^: that is, relative rate of hydride/deuteride transfer catalysed by the *Thermotoga maritima* DHFR [8], which shows temperature-independent behaviour below 25°C, and temperature-dependent behaviour (Δ*E*_a_ = *E*_a_^D^ – *E*_a_^H^ = 2.5 kJ mol^−1^) above this ‘breakpoint’ temperature.

This is then substituted into the Eyring equation, with this approach termed ‘macromolecular rate theory (MMRT)’[7]; *T*_0_ is an arbitrary reference temperature and $\Delta C_{p}^{‡}$ is the difference in heat capacity between the reactant and transition states, which is generally observed to be negative (figure 1) [9–11].

Enzyme kinetics are often measured using steady-state methods, with initial velocities analysed using Michaelis–Menten theory [12]. However, for more complex multi-step mechanisms, interpretation of these kinetics can be difficult as the rate-limiting step may not be well defined. Rapid-mixing methods, such as stopped-flow can provide additional information on these reactions (e.g. substrate-binding kinetics) [13–16], but the use of these methods is fairly limited. KIEs (e.g. *k*_H_/*k*_D_) have also become established as a powerful method of augmenting enzyme kinetic analysis, particularly for reactions involving partially rate-limiting H-transfer steps [17–20]. At the turn of the twenty-first century, examples of enzyme reactions with anomalous KIEs began to emerge (figure 1) [8,21–28]. In some cases, these studies measured the temperature dependence of KIEs and found examples where the temperature dependences fell outside of the values expected from semi-classical kinetic models, such as the Bell correction model [29]. These data were rationalized by invoking a quantum mechanical tunnelling contribution to the H-transfer (sometimes described by *κ* in equation (2.1) [30,31]), with unusual temperature dependencies arising from the environmental coupling of protein motions to the reaction coordinate [20,32–34]. Since then, many examples of H-transfer enzymes that exhibit anomalous KIEs have emerged; e.g. those with KIEs much larger than the semi-classical limits [21,23,24,27,28] and/or unusual temperature dependencies [8,21–26].

Since our Millennium publication [6], a range of additional experimental probes of tunnelling and dynamics during enzyme-catalysed H-transfer reactions have also emerged. The effect of hydrostatic pressure on these reactions has been explored [35–39], but the experiments are difficult to perform and interpretation of the effects is complicated, as the effect of increasing pressure on the structure of enzymes appears to be anisotropic [39]. Changes in solution viscosity on these reactions have similarly been investigated [40–42], with examples of both viscosity-dependent and -independent KIEs reported.

In addition to KIE measurements, isotopic labelling has long been used as a (bio)physical probe, included to probe enzyme dynamics using infrared and vibrational Stark spectroscopies [43,44], as well as through multidimensional nuclear magnetic resonance spectroscopy [45,46]. A more direct probe of protein, cofactor and/or substrate vibrations is the ‘heavy enzyme’ effect, measured using proteins or substrates that have been isotopically labelled with ^15^N, ^13^C and/or ^2^H [9,11,47–52]. A range of behaviours has been observed in these experiments, with notable examples showing specific vibrations in individual amino acids [47] or the substrate [9] that appear to couple to the H-transfer step. In concert with the experiment, computational studies have identified vibrations/motions of the active site, second sphere and non-active site residues that appear to couple to the reaction coordinate [33,34,53–56] and/or facilitate the sampling of multiple populations of the enzyme [57–59]. The importance of coupled vibrations in enzyme catalysis has been contested [58,60] but such networks have since been identified using hydrogen/deuterium exchange (HDX)-mass spectrometry in a number of ‘H-tunnelling’ enzymes [61–63]. An alternative approach is to use (ultrafast) spectroscopy to experimentally probe fast vibrations/dynamics. This allows access to sub-ps timescales. Such experiments also provide important information about the initial photochemical events in photocatalysts (see below), but can also be used to probe the coupling of protein vibrations to the substrate and/or coenzyme by using mutagenesis and/or selective isotopic labelling with infrared detection [11,64–69]. In addition, with recent advances in computational methods, it is now possible to practically model excited-state (photo)chemistry in photoreceptors and photoenzymes using quantum mechanics and quantum mechanics/molecular mechanics methods [ 70–74].

## 3. Photocatalysis—the new paradigm for enzyme action

Despite major advances in our mechanistic understanding of enzyme catalysis made so far in the twenty-first century, assessing how structural transitions and dynamics influence function remains arguably as one of the leading unmet challenges in understanding enzyme function. Ultimately, this goes to the core of any ambition to predictively create bespoke catalysts for widespread applications. However, as almost all enzyme-catalysed reactions are thermally activated, studies of dynamical processes that facilitate and/or couple to catalysis are limited by diffusion-associated processes that are almost impossible to monitor experimentally. Consequently, the reaction chemistry can only easily be studied across relatively long milliseconds–seconds timescales and over a small range of temperatures. Conversely, for light-activated enzymes or photoenzymes, diffusion-associated components can be completely removed from experimental kinetic analyses and it is possible to trigger catalysis by using a single pulse of light [75]. This facilitates a more detailed analysis of catalytic mechanism over a wide range of temperatures and timescales (femtoseconds–seconds) in a way that is generally inaccessible with thermally activated enzymes. Moreover, these systems open up the possibility of time-resolved structural analysis using pulsed light activation to provide a time-resolved series of snapshots or movies of enzyme catalysis, a grand challenge for enzymology [76]. As such, photoenzymes offer a unique opportunity to provide a complete molecular description of catalysis and to investigate the role of fast motions coupled to the reaction chemistry [77–80]. However, natural photoenzymes are rare. Apart from the photosynthetic reaction centres, only three photoenzymes have been discovered to date, namely, protochlorophyllide oxidoreductase (POR), DNA photolyase and fatty acid photodecarboxylase (FAP) [75]. In POR, catalysis is triggered by the excitation of the substrate itself, whereas DNA photolyase and FAP both exploit the inherent photochemical reactivity of a flavin cofactor (flavin adenine dinucleotide, FAD) to drive reaction chemistry.

### (a) POR

**Figure 2.**
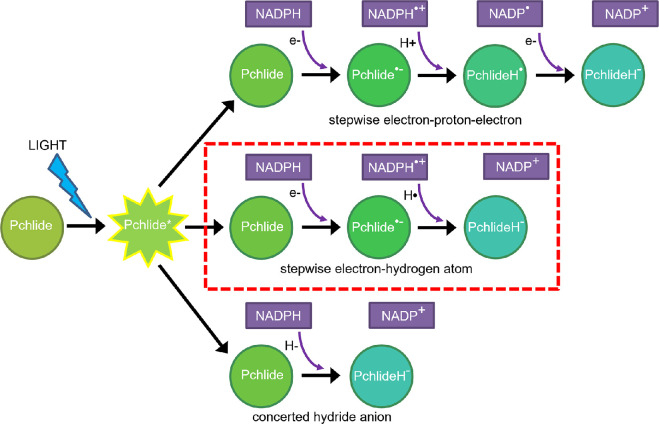
The multi-step nature of the hydride transfer chemistry in POR. Three possible routes for enzymatic hydride transfer reactions from NAD(P)H coenzyme to substrate are shown. The stepwise transfer of an electron to the excited state of the Pchlide substrate followed by H-atom transfer (shown in red box) was identified by experimental and computational approaches [86,90].

The light-driven chlorophyll biosynthetic enzyme POR catalyses the *trans* addition of hydrogen (two protons and two electrons) across the C17–C18 double bond of the chlorophyll precursor, protochlorophyllide (Pchlide) and is an essential enzyme for the development of the photosynthetic apparatus in plants [81]. Due to its unique requirement for light it is also an important model system for studying mechanisms of enzymatic H-transfer reactions [82]. Recent breakthroughs in biophysical, structural and computational studies of POR, have facilitated a detailed understanding of the POR photocatalytic cycle and the associated reaction dynamics [41,67,83–92]. Upon illumination the Pchlide substrate acts as the light-sensing chromophore, where excited-state charge separation [67] triggers two sequential enzymatic H-transfer reactions that proceed via quantum mechanical tunnelling on the microsecond timescale [85]. It is likely that the role of light in POR photocatalysis is to overcome the barrier for the energetically unfavourable hydride anion (two electrons and a proton) transfer from NADPH to the Pchlide molecule, which occurs in approximately 500 ns to generate a negatively charged Pchlide anion intermediate (Pchlide H^−^) [85,93]. Although enzymatic hydride transfers are widely thought to proceed as a single chemical entity, the hydride transfer reaction in POR occurs in a multi-step process (figure 2) [86,90]. The identification of this stepwise hydride transfer is only possible to observe in POR due to the very fast time resolution that comes from initiating reaction chemistry with short laser pulses, again demonstrating the advantage of using photoenzymes to study enzyme mechanisms. In this case, hydride involves an initially excited-state electron transfer from NADPH to Pchlide followed by a proton-coupled electron or H-atom transfer [86]. The multi-step nature of the hydride transfer chemistry was confirmed by computational studies, which also showed that the excited-state electron transfer from NADPH to the Pchlide facilitates a subsequent hydrogen atom transfer by weakening the C–H bond of NADPH that is to be broken [90]. Following the hydride transfer chemistry in POR the reduction of the C17–C18 bond of Pchlide is completed by a proton transfer step on the microsecond timescale, either directly from a conserved active site residue (recently proposed to be a Cys residue) or via active site water molecules [85,92]. This proton transfer step is reliant on solvent-coupled protein dynamics although the exact mechanism of the protonation reaction remains unknown [41,92]. It should also be noted that a recent computational study proposed an alternative mechanism for the POR reaction, whereby Pchlide photochemistry initiates electron transfer from a conserved Tyr residue to the C18 position of Pchlide. This is suggested to lead to a subsequent proton transfer, also from the Tyr residue, followed by an electron and hydride transfer from NADPH, although there is currently no experimental evidence for this mechanism [91].

Ultimately, detailed insight into how light energy is harnessed by POR to drive the reaction chemistry is only possible with a structural understanding of the POR–NADPH–Pchlide ternary complex. This had remained elusive for many years, but crystal [87,88] and electron microscopy [89] structures of the enzyme have recently been solved. The crystal structures of cyanobacterial PORs in the presence and absence of bound NADPH revealed a typical Rossmann-fold with a central β-sheet surrounded by multiple flexible loops [87,88]. Although different binding modes for the Pchlide substrate have been proposed based on computational modelling and cryo-electron microscopy studies [87,89] it appears that the reaction mechanism is most compatible with the structural model derived from cryo-electron microscopy [92]. The Pchlide binds in a deep active site pocket with polar functional groups forming hydrogen bonds with key residues in the enzyme active site [87,89,92]. A ‘lid’ which comprises two conserved loop regions of the protein closes over the hydrophobic edge of the Pchlide molecule, which ensures the Pchlide is in an optimal geometry for efficient photocatalysis [94]. This positions an active site cysteine close to the C17–C18 double bond of Pchlide, allowing it to participate in both the hydride and proton transfer reactions, while a conserved glutamine is likely to interact with the central Mg atom of the substrate and tune its electronic properties for efficient photochemistry [92]. The movement of the two flexible lid regions of POR over the Pchlide substrate also leads to larger-scale conformational change in the protein that triggers oligomer formation [89,94]. These POR oligomers adopt helical filaments with the lipid bilayer that shape the photosynthetic membrane for chlorophyll synthesis. The oligomeric structure of POR may also lead to increased photocatalytic efficiency by facilitating energy transfer between neighbouring Pchlide molecules [89].

### (b) DNA photolyase

**Figure 3.**
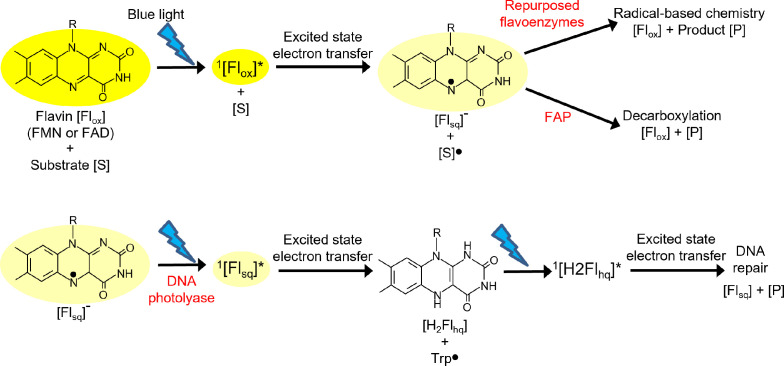
Potential photochemical pathways catalysed by flavoenzymes upon illumination with blue light, which catalyse the conversion of a substrate (S) to product (P). Three possible routes for photocatalysis are shown upon excitation of either oxidized flavin (Fl_ox_) or the one electron-reduced semiquinone (Fl_sq_) by DNA photolyase, FAP or repurposed flavoenzymes (e.g. ‘ene’ reductases or monooxygenases). Excitation of Fl_ox_ leads to electron abstraction to form Fl_sq_ and excitation of Fl_sq_ abstracts an electron to form the fully reduced hydroquinone state (Fl_hq_).

DNA photolyase uses blue light to catalyse the repair of pyrimidine dimers in DNA caused by UV damage [95]. They are structurally homologous to cryptochrome photoreceptors and both classes of protein share similar electron transfer pathways upon excitation of a flavin cofactor. The photochemical repair mechanism has been elucidated by detailed biophysical and computational studies combined with X-ray crystallographic structures [96,97]. The damaged DNA binds to the enzyme by ionic interactions, which results in the pyrimidine dimer flipping out to bring it in close proximity to the FAD within the active site cavity [98]. The reaction involves multiple redox steps that lead to a splitting of the cyclobutane ring. The FAD cofactor abstracts an electron from a nearby tryptophan residue within 1 ps, either upon direct excitation or via energy transfer from an additional light-harvesting antenna cofactor, methenyltetrahydrofolate, which allows a photoresponse across a wider range of the visible spectrum to improve photochemical efficiency [96,97]. This triggers a series of electron-transfer reactions along a pathway that involves a further three tryptophan residues to generate a radical pair between the flavin semiquinone and a tryptophanyl radical. This photoreduction is repeated a second time to form the reduced, anionic hydroquinone state (FADH**^–^**), which is the active species that is capable of repairing the DNA lesion (figure 3) [99].

Advances in time-resolved serial crystallography measurements have now allowed the structural changes associated with photocatalysis by DNA photolyase to be elucidated across multiple timescales and have begun to uncover how DNA photolyases repair DNA in real time at the atomistic level [77–79,100]. In the first of these studies, it was shown that the FAD twists on the sub-microsecond timeframe and is stabilized by an Asn/Arg-Asp redox sensor triad in the enzyme active site [100]. Further time-resolved serial crystallography measurements using nanosecond laser excitation have also shown that the FAD excited state exists in a highly bent geometry. Electron transfer from the flavin excited state to the damaged DNA triggers the repair reaction on the picosecond-to-nanosecond timescale, which involves the lysis of two covalent bonds via a single-bond intermediate [77,78]. DNA repair is then followed by recovery of the enzyme active site and the FADH**^−^** cofactor to their respective resting-states within 500 ns. Two further steps then occur on a much slower microseconds timescale, involving sequential product release, with the 3′-thymine migrating out of the active site prior to the 5′-base, and restoration of the repaired DNA double-helix structure [77,78]. It has also now been possible to capture structural snapshots that cover the earliest femto and picosecond timescales, which highlight structural changes that occur upon electron transfer from the FAD cofactor along the tetrad of tryptophan residues [79]. These show distinct conformational rearrangements of active site side chains and waters, including a nearby methionine residue, from 1 to 20 ps that accompany charge separation close to the second tryptophan, and further changes from 20 ps around the fourth tryptophan residue. Importantly, these time-resolved crystallographic studies of photolyase, enable direct observation of enzymatic catalysis at work, at atomic resolution, across a wide time range that spans picosecond to microsecond timescales, and include electron transfer, bond breaking and product migration events [77–79,100].

### (c) FAP

The other flavin photoenzyme that exists in nature, FAP, was only discovered in 2017 in microalgae [101]. FAP is a member of the glucose–methanol–choline oxidoreductase family and uses a FAD cofactor to catalyse the decarboxylation of mid- to long-chain fatty acid substrates to the corresponding alkane in response to blue light. This ability to decarboxylate fatty acids in a light-dependent manner has led to FAP becoming an exploitable photobiocatalyst, and over the last several years FAP enzymes have been employed in various biocatalytic cascades and synthetic biology applications [102]. The wild-type FAP enzyme, which prefers long-chain fatty acids as its native substrate, has been used, in some cases, in conjunction with additional enzymes, to produce long-chain alkanes, secondary fatty alcohols, long-chain aliphatic amines, esters and epoxides [103–106]. The substrate scope of FAP has also been altered, either by employing decoy molecules to limit the unoccupied space available in the active site or by using rational protein engineering and directed evolution approaches to produce new photobiocatalysts. These approaches have led to the production of short-chain alkanes, selective conversion of α-functionalized acids and *trans* fatty acids, deuteration of various carboxylic acids and the production of ethylbenzene [107–112]. However, one of the major drawbacks of using FAP for such biocatalytic applications has been its susceptibility to photoinactivation, especially in the absence of bound substrate. This inactivation has been linked to the formation of reactive oxygen species following the reaction with the triplet state of the FAD cofactor, which may lead to radical formation and damage of active site residues [113,114]. A more in-depth molecular basis for these ‘off pathway’ radical escape and enzyme inactivation processes will be required for further exploitation of FAP as an industrial photobiocatalyst.

A detailed understanding of the mechanism of the light-driven photodecarboxylation chemistry catalysed by FAP has been established through time-resolved and cryogenic spectroscopy measurements, computational chemistry approaches and both static and serial crystallography studies [80,101,115,116]. The enzyme contains an N-terminal FAD-binding domain and a C-terminal substrate-binding domain, where hydrophobic residues within a long substrate channel interact with the fatty acid substrate to ensure that the carboxylate moiety is located close to the FAD cofactor [101]. A number of highly conserved active site residues are located in close proximity to the FAD cofactor and cause it to adopt a ‘butterfly bent’ conformation of 14−18^o^. This is suggested to affect the energy levels of the flavin and lead to a red-shift in its absorbance features that may enhance its light-harvesting properties [80]. Upon excitation with blue light the singlet excited state of the FAD rapidly (300 ps) abstracts an electron from the carboxylate group of the fatty acid substrate to form a biradical state consisting of a fatty acid radical and an anionic FAD semiquinone (figure 3). This initial excited-state electron transfer is an important step in catalysis, leading to the decarboxylation of the fatty acid anion to form an alkyl radical in an energetically, barrier-less process [80,116]. Further proton and electron transfer steps then occur to form the final alkane product via an unusual, red-shifted FAD intermediate. Although the exact mechanism of these slower steps in the reaction cycle is unclear, strictly conserved active site Cys and Arg residues have been shown to be important [80,115]. The chemical nature of the red-shifted flavin intermediate is also unknown but may be caused by interactions of the oxidized form of the cofactor with negatively charged species (e.g. Cys thiolate, bicarbonate), deprotonated active site residues or active site water molecules. The catalytic cycle is completed by a proton or hydrogen atom transfer, allowing the release of the alkane product and CO_2_ (or bicarbonate), which facilitates the binding of a new fatty acid substrate to the resting form of the enzyme to initiate a new catalytic cycle [80,115].

## 4. Non-natural photoenzymes

The recent emergence of photobiocatalysis to enable chemical reactions that proceed through reactive and transiently populated species is beginning to open up photobiocatalytic routes to chemical synthesis [117]. The ability of some biological chromophores to participate in excited-state electron transfer reactions has now been utilized to catalyse radical-based chemistry that does not normally occur in nature and is often challenging using small-molecule catalysts. This was initially demonstrated in photoreduction chemistry by nicotinamide cofactors upon illumination of the cofactor or substrate when repurposing nicotinamide-dependent enzymes to catalyse radical dehalogenation reactions [118,119]. Since then, the well-established excited-state properties of flavin cofactors (FMN and FAD) to undergo photoreduction upon illumination have been widely exploited as a light-sensitive chromophore in numerous repurposed photoenzymes (figure 3). This has involved using native and engineered ‘ene’-reductase and monooxygenase flavoenzymes as photobiocatalysts to access new chemical reactions, including reactions important to chemical industries such as radical-based cyclization, alkylation, cross-electrophile coupling, hydrodehalogenation, reduction and polymerization reactions [120–130]. However, the quantum yield and turnover numbers for these non-natural flavin-based photoenzymes are generally poor and the excited state of the flavin cofactor is often short-lived due to rapid quenching by the protein scaffold [117]. The photochemical efficiency of these systems has been improved by the conjugation of highly absorbing dyes to act as light-harvesting antennae [131] or the addition of an exogenous photo-oxidant to trigger radical formation within the enzyme’s active site [130]. Nonetheless, a deeper understanding of the structures and reaction mechanisms of this exciting array of non-natural photoenzymes is required to support the rational design of highly efficient photoenzymes and to open up a range of new radical-based chemistries.

**Figure 4.**
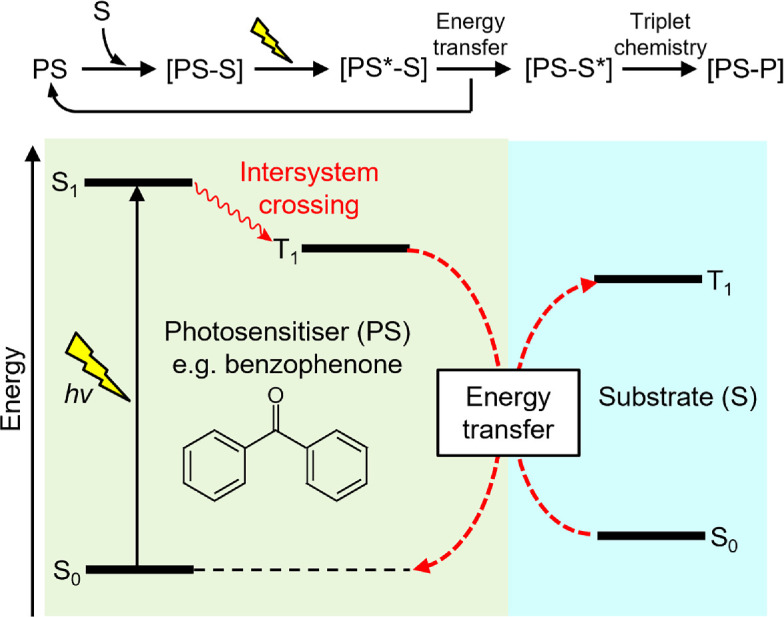
Jablonski diagram describing the mechanism of photocatalysis by recently identified triplet energy transfer photoenzymes [136,137]. A photosensitizer (PS), which is benzophenone in this case, is excited into a singlet state (S_1_). This subsequently undergoes intersystem crossing to a triplet state (T_1_) with a high quantum yield. The triplet energy is transferred from the PS to the substrate (S), which then undergoes transformation to a product state (P).

All of the above natural and repurposed photoenzymes use excited-state electron transfer mechanisms to trigger photocatalysis upon absorption of light. However, the harnessing of visible light to access excited triplet states of organic chromophores has also been widely employed by chemists to catalyse a variety of unique synthetic transformations [132,133]. Moreover, the ability to now introduce new functional elements into proteins beyond those found in nature through genetic code expansion methodology allows the incorporation of non-canonical amino acid side chains into enzyme active sites in a site-selective manner [134,135]. To this end, these two approaches have recently been combined to incorporate the genetically encoded triplet photosensitizer benzophenone into the active site of either a de novo Diels–Alderase or the multi-drug-resistance regulator LmrR (figure 4) [136,137]. By coupling this to multiple rounds of enzyme evolution, new efficient and enantioselective photoenzymes have been produced, which can use triplet energy transfer to catalyse intramolecular and bimolecular cycloadditions that are difficult to achieve selectively with small-molecule catalysts. A similar approach has also been used to generate a genetically encoded photosensitizer protein that is capable of reducing many CO_2_-reducing catalysts via a long-lived triplet excited state [138]. In addition, the same photosensitizer protein has been employed to catalyse a dehalogenation reaction via triplet sensitization of a Ni(II) cofactor upon illumination of the benzophenone chromophore [139]. These studies all demonstrate the potential of introducing new modes of catalysis into enzymes and suggest that the incorporation of alternative non-canonical amino acid residues may lead to the development of a new generation of photoenzymes that can catalyse a range of excited-state chemistries.

## 5. Concluding remarks

We are on the cusp of exploiting the natural photochemistry of flavin to radically extend existing capabilities in enzyme engineering and design through the emergence of a new field, namely, photobiocatalysis. This field will open up new high-energy reaction pathways while maintaining the beneficial, highly selective specificities of thermally activated enzymes to guide target substrates to the flavin cofactor contained in bespoke photocatalysts through top-down enzyme engineering (or enzyme repurposing). More ambitiously, bottom-up protein de novo design can also deliver first-of-a-kind photobiocatalysts in which the flavin (or other light-sensitive group) is embedded within protein or peptide scaffolds, consistent also with designs that might incorporate an expanded amino acid alphabet. The ambition must be to *generalize* the design and engineering of photobiocatalysts, to give broad access to difficult-to-achieve reactions, through protein engineering and de novo design. In this way, delivery of first-of-a-kind flavin-based photoenzymes should bring photobiocatalysis into the biological mainstream and expand reaction space well beyond that available with natural enzymes.

## Data Availability

This article has no additional data.
